# Two-dimensional cell membrane chromatography guided screening of myocardial protective compounds from Yindan Xinnaotong soft capsule

**DOI:** 10.1186/s13020-024-01046-1

**Published:** 2025-01-04

**Authors:** Si-Min Shao, Xuan Ji, Xing Wang, Run-Zhou Liu, Yu-Ru Cai, Xiaobing Lin, Ze-Jie Zeng, Ling Chen, Liu Yang, Hua Yang, Wen Gao

**Affiliations:** State Key Laboratory of Natural Medicines, School of Traditional Chinese Pharmacy, China Pharmaceutical University, No. 639 Longmian Road, Nanjing, 211198 China

**Keywords:** Cell membrane chromatography, Imidazole-modified silica gel, Yindan Xinnaotong soft capsule

## Abstract

**Background:**

Cell membrane chromatography (CMC) is a biochromatography with a dual function of recognition and separation, offering a distinct advantage in screening bioactive compounds from Chinese medicines (CMs). Yindan Xinnaotong soft capsule (YD), a CM formulation, has been widely utilized in the treatment of cardiovascular disease. However, a comprehensive mapping of the myocardial protective active compounds remains elusive.

**Purpose:**

To establish a stable and efficient 2D H9c2/CMC-RPLC-MS system, and to utilize it for screening the active compounds of YD that are associated with myocardial protection.

**Methods:**

An imidazole-modified silica gel exhibiting high modification efficiency and protein binding capacity was synthesized to enhance the longevity and efficiency of H9c2/CMC. Subsequently, the potentially bioactive compounds of YD were screened by integrating the 2D H9c2/CMC-RPLC-MS system with a high-content component knockout strategy. Additionally, an RNA-seq approach was employed to predict the targets and mechanisms of YD and the active compounds for myocardial protection.

**Results:**

The developed imidazole-modified H9c2/CMC exhibits remarkable selectivity, specificity, stability, and reproducibility. Following three rounds of screening, a total of 24 potential myocardial protective compounds were identified, comprising 8 flavonoids, 8 phenolic acids, 4 saponins, and 4 tanshinones. Bioinformatic analysis utilizing RNA-seq indicated that the FOXO signaling pathway, with FOXO3 identified as a key target, plays a significant role in the cardioprotective effects of YD. Furthermore, all 24 screened compounds exhibit strong binding affinities with FOXO3 evaluated by molecular docking.

**Conclusion:**

A highly stable and efficient 2D imidazole-modified H9c2/CMC-RPLC-MS system was developed, allowing for the screening of potentially active compounds from YD. Through the integration of the bioinformatic analysis, the pharmacodynamic foundation of YD for myocardial protection has been comprehensively characterized.

**Graphical Abstract:**

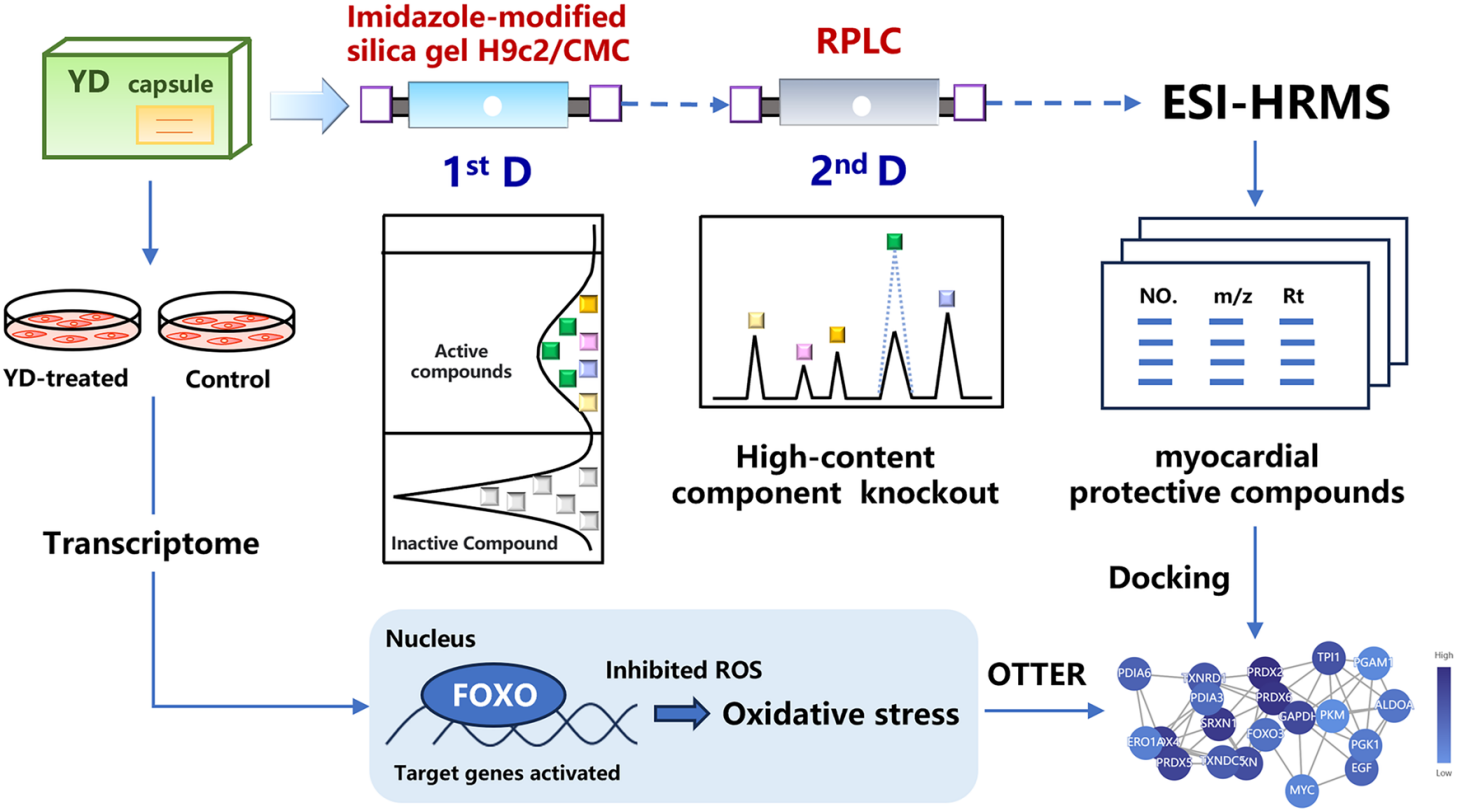

**Supplementary Information:**

The online version contains supplementary material available at 10.1186/s13020-024-01046-1.

## Introduction

Cell membrane receptors are specialized molecules characterized by high-affinity, and saturable molecules, situated on the cell surface. Cell membrane chromatography (CMC) is a biomimetic chromatographic technique that immobilizes the membrane receptors onto a silica carrier, effectively simulating the interactions between cell membrane receptors and ligands in vivo within a chromatographic framework. As the herbal extract traverses through the CMC column, compounds exhibiting high affinity are retained, while nonspecific analytes are rapidly eluted. Therefore, CMC possesses dual functionalities of biological recognition and chromatographic separation characteristics [1, 2], offering a distinct advantage in the screening of bioactive compounds from Chinese medicines (CMs). Additionally, an online screening system that integrates two-dimensional liquid chromatography (2D-LC) with CMC improves the screening efficiency of complex matrices [3]. This integrated approach initially captures potential active compounds in the first dimension, CMC, followed by the analysis of the retention fractions using the second-dimensional reverse phase liquid chromatography (RPLC), ultimately identifying by the high-resolution mass spectrometry.

The stability of the cell membrane stationary phase (CMSP) is fundamental to the concept of CMC. Traditionally, the adhesion force that binds membranes to silica gel primarily depends on hydrophobic interactions [1]. However, this model has posed challenges, leading to membrane degradation and detachment, resulting in a shortened column lifespan and reduced reproducibility [4]. Recently, the development of carbon–nitrogen bonds between amino groups on proteins and the silica gel substrate offers a promising alternative for the preparation of CMSP. Specifically, compounds such as 1,1-carbonyldiimidazole (CDI), glutaraldehyde (GA), and (3-Glycidyloxypropyl) trimethoxysilane (GLYMO) [5–7] have been reported as optimized carriers in this regard. However, in addition to proteins, cell membranes are rich in phospholipids [7–9], including phosphatidyl-ethanolamine and phosphatidyl-serine, which contain free amino groups. As a result, the interaction between cell membranes and CDI-modified silica gel is expected to be enhanced due to the formation of covalent bonds, rather than solely relying on hydrophobic interactions.

Yindan Xinnaotong soft capsule (YD) is a well-known CM with a formulation comprised of eight herbs (Ginkgo Folium, Salviae Miltiorrhizae Radix et Rhizoma, Erigerontis Herba, Crateagi Fructus, Notoginseng Radix et Rhizoma, Gynostemma pentaphyllum, Allii Sativi Bulbus, and Borneolum) [10]. Recent studies have found that YD exhibits anti-inflammatory and oxidative stress activities, and has gained widespread acceptance in the treatment of cardiovascular diseases [11, 12]. However, a comprehensive mapping of the myocardial protective active compounds remains elusive.

**Fig. 1:**
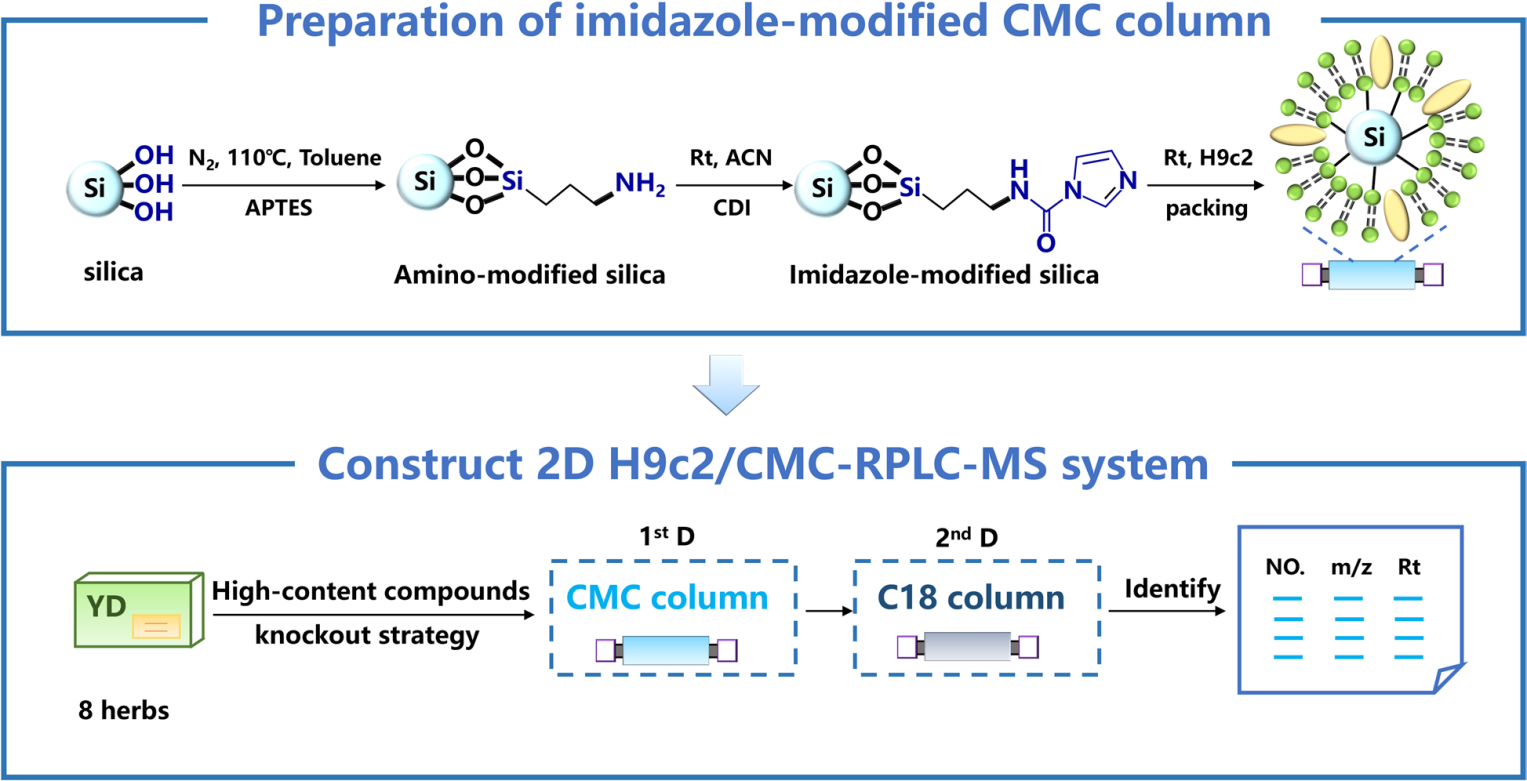
Imidazole-modified H9c2/CMC preparation and established the 2D H9c2/CMC-RPLC-MS system to screen active compounds from YD

In this study, we synthesized an imidazole-modified silica gel characterized by high modification efficiency and notable protein binding capacity. Utilizing this modified silica gel, we successfully constructed an H9c2/CMC with an extended lifespan and improved efficiency. Subsequently, the 2D H9c2/CMC-RPLC-MS system was established. Notably, the cell membrane retains a limited number of receptors for screening affinity compounds. The presence of high-concentration or high-affinity compounds often results in column overload, which hinders the screening process [13, 14]. Therefore, we applied a slightly modified version of our previously developed high-content compounds knockout strategy [14] to screen the active compounds of YD for their myocardial protective effects (Fig. 1). By integrating bioinformatics analysis and molecular docking, the key targets and mechanisms of YD and its active compounds in relation to myocardial protection were investigated. This strategy allowed us to effectively identify and evaluate the active compounds for CMs, ensuring the reliability and accuracy of our findings.

## Materials and methods

### Materials and reagents

YD sample (batch no. 20201045) was purchased from Guizhou Bailing Group Pharmaceutical Co., Ltd. (Guiyang, China). Seven intermediates under the preparation procedure of YD [10], including extract of Ginkgo Folium (GF), ethanol extract of Salviae Miltiorrhizae Radix et Rhizoma (ESM), water extract of Salviae Miltiorrhizae Radix et Rhizoma (WSM), mixed extract of Notoginseng Radix et Rhizoma, Crateagi Fructus, and Gynostemma pentaphyllum (Mix), water extract of Erigerontis Herba (EH), Borneolum (BN), oil of Allii Sativi Bulbus (AS) were offered from Guizhou Bailing Group Pharmaceutical Co., Ltd. (Guiyang, China).

The reference standard of scutellarin, ginsenoside Rd, salvianolic acid B, kaempferol-3-*O*-2ʺ-(6ʺ-p-coumaroyl) glucosyl rhamnoside, and ginsenoside Rb1 were purchased from Chengdu Must Bio-technology Co., Ltd (Sichuan, China). Dexamethasone acetate (DXM) and propafenone hydrochloride (PPH) were purchased from Macklin Co. (Shanghai, China). The purity of all reference standards was not less than 98%. Silica gel (5 μm, 100 Å) was purchased from Bonna-Agela Co. (Tianjing, China) and was activated at 105 ℃ for 120 min before use.

The LC/MS-grade acetonitrile and HPLC-grade methanol were purchased from Merck (Darmstadt, Germany). Deionized water (18 MΩ·cm) was purified by a Milli-Q water purification system from Millipore (Bedford, MA, USA). The HPLC-grade ammonium acetate was purchased from ROE Scientific Inc. (USA). Dulbecco’s modified Eagle’s medium (DMEM) and phosphate buffered saline (PBS) were purchased from Nanjing Keygen Biotech Co. (Nanjing, China). Fetal bovine serum (FBS) and trypsin were purchased from Gibco Life Technology Co. (Australia).

### Preparation of standard and sample solutions

Standard stock solutions of DXM, PPH, scutellarin, ginsenoside Rd, salvianolic acid B, kaempferol-3-*O*-2ʺ-(6ʺ-p-coumaroyl) glucosyl rhamnoside, and ginsenoside Rb1 were prepared in 75% methanol and stored at 4 ℃ until use.

YD was prepared according to previously reported methods [15]. The outer capsule layers of YD were removed, and the contents were exactly weighed (approximately 0.6 g) and put into a 100 mL conical flask, sonicated for 30 min at 100 Hz with 60 mL of methanol/water solution (75:25, *v/v*). After cooling at room temperature, 75% methanol was added to compensate for the lost weight.

The K_*(x)*_YD samples were prepared by mixing the intermediates, excluding one specific intermediate *x* each time, i.e. K_*(GF)*_YD, K_*(SM)*_YD, K_*(Mix)*_YD, K_*(EH)*_YD, K_*(AS)*_YD, and K_*(BN)*_YD, respectively. Both the K_*(x)*_YD samples and the individual intermediates were extracted in the same procedures as YD.

### Cell culture

H9c2 rat cardiac myoblasts were obtained from the Cell library of typical culture preservation committee of Chinese Academy of Sciences (Shanghai, China) and were cultured in DMEM medium supplemented with 10% (*v/v*) FBS, 80 U mL^−1^ penicillin, and 80 μg mL^−1^ streptomycin in a humidified atmosphere of 5% CO_2_ at 37 ℃. For the preparation of the H9c2/CMC model, cells from exponentially growing cultures were employed.

Trypsin was used to collect the H9c2 cells and then centrifuged at 110 *g* for 10 min after being washed three times with PBS (pH 7.4). An ultrasonic cell disruptor (Emerson Electric Co., USA) was used to rupture the cell suspension in PBS at 75 W, 2 s, 5 times, and 20 s intervals [16]. After centrifuging the resultant homogenate at 1000 *g* for 10 min, the supernatant was centrifuged at 12,000 *g* for 20 min and stored at –80 ℃ until use.

### Preparation of imidazole-modified H9c2/CMC columns

The reaction scheme is shown in Fig. 1. First, (3-aminopropyl)-triethoxysilane (APTES) (Macklin Co., Shanghai, China) was used to add amino groups to the surface of the silica gel. In a nutshell, 40 mL of toluene was combined with 1.0 mL of APTES and 1.0 g of degassed silica gel, and heated at 110 °C for 12 h. Following drying, the sample was combined with 1 g of CDI (Macklin Co., Shanghai, China) in 40 mL of acetonitrile, and shaken for 12 h at room temperature to allow the imidazole to bind with amino groups. At last, the other end of imidazole would be able to link to cell membranes by reacting with the abundant amino groups on the membranes after 5 min of vortex in a vacuum and 24 h of incubation at 4 °C.

After incubation, the imidazole-modified CMSP was washed three times with PBS by centrifuging at 110 g for 5 min. The pellet was suspended in PBS and packed into the column (10 mm × 2 mm I.D.) by a column packing machine (Dalian Replete Science and Technology Co., China) [3]. Then, the column was equilibrated with 5 mM ammonium acetate at 0.2 mL min^−1^ until the baseline and column pressure were stable. The blank control was prepared without a cell membrane by the same method.

### 2D H9c2/CMC-RPLC-MS condition

The first-dimension columns were CMC columns, and the analysis was performed on the Waters ACQUITY H-Class system. The mobile phase was 5 mM ammonia acetate in water, the flow rate was 0.2 mL min^−1^, the sample injection volume was 10 μL, and the UV wavelengths of VWD were 310 nm and 245 nm. The components that were kept in the first dimension will subsequently be analyzed in the second dimension.

For the second-dimension separation, an Agilent ZORBAX Eclipse Plus C18 column (2.1 × 150 mm, 1.8 μm) was used. The mobile phase consisted of acetonitrile (A) and 0.1% aqueous formic acid (B) with the following gradient elution: 0 min, 5% A; 6 min, 12% A; 9 min, 14% A; 27 min, 27% A; 40 min, 55% A; 58 min, 100% A; 60 min, 100% A. The flow rate was 0.3 mL min^−1^. The column temperature was kept at 37 ℃.

The I-Class Xevo G2-XS QTOF mass spectrometer (Waters, Milford, USA) with an electrospray ion source was operated in both positive and negative scanning modes. The ion source parameters were set as follows: capillary voltage, 4 kV; sample cone, 40 V; source and desolvation temperatures, 100 ℃ and 400 ℃, respectively; and desolvation and cone gas flow, 800 L h^−1^ and 50 L h^−1^, respectively. The data-independent acquisition mode was adopted, the scanning range was *m/z* 100–1500 Da, and the collision energy was set at 30 eV and 60 eV. Data acquisition and processing were conducted using MassLynx 4.1 software (Waters, Milford, USA).

### Transcriptome and data processing

Gene profiling was performed by BGI Genomics Co., Ltd, using an Affymetrix Human Genome U133 Plus 2.0 array (Affymetrix; Thermo Fisher Scientific, USA). Briefly, total RNA was extracted from YD-treated (50 μg/mL, 12 h) MCF-7 and control groups with Trizol reagent (Invitrogen, USA) according to the manufacturer’s protocol and purified with GeneChip IVT Express Kits (Affymetrix, 901, 229). Then, the GeneChip Hybridization, Wash, and Stain Kit (Affymetrix, 900, 720) was used following the manufacturer’s instructions. Slides were scanned by a GeneChip Scanner 3000 (Affymetrix) and an Affymetrix GeneChip Command Console 4.0 with default settings. Genes that were differentially expressed between YD-treated and control groups using DESeq2, with a q-value < 0.05, were set as the threshold for significantly differential expression. The raw sequencing data was uploaded to the NCBI SRA (http://www.ncbi.nlm.nih.gov/sra) with the accession number PRJNA1186569.

### Molecular docking assay

The X-ray crystal structure of protein FOXO3 (PDB code: 2K86) was downloaded from the Protein Data Bank database (https://www.rcsb.org/) and processed in PyMOL (Version 2.5.0). Crystal waters and cocrystallized ligands were removed, and hydrogen atoms were added to the protein according to the protonation states of the chemical groups at pH 7.0. The structures of the 24 compounds and quinidine were collected from PubChem (https://pubchem.ncbi.nlm.nih.gov/) and optimized to minimize energy using ChemBio3D (version 14.0). Applying the Lamarckian genetic process and AutoDock (Version 1.5.7), 24 compounds and quinidine were docked with FOXO3.

### Cellular thermal shift assay

H9c2 protein solutions was pretreated with the YD solutions (50 μg/mL) and high-score molecular docking compounds (YD-1: 10 μM, YD-2: 10 μM, C-2: 10 μM) or DMSO at room temperature. Subsequently, the solution was divided into equal parts in tubes and heated at different temperatures (47 ℃, 50 ℃, 53 ℃, 56 ℃, 59 ℃, 62 ℃) for 3 min. After centrifugation, the supernatants were added with loading buffer, followed by western blotting experiment.

## Results and discussion

### Synthesis and characterization of covalently modified silica gel

**Fig. 2:**
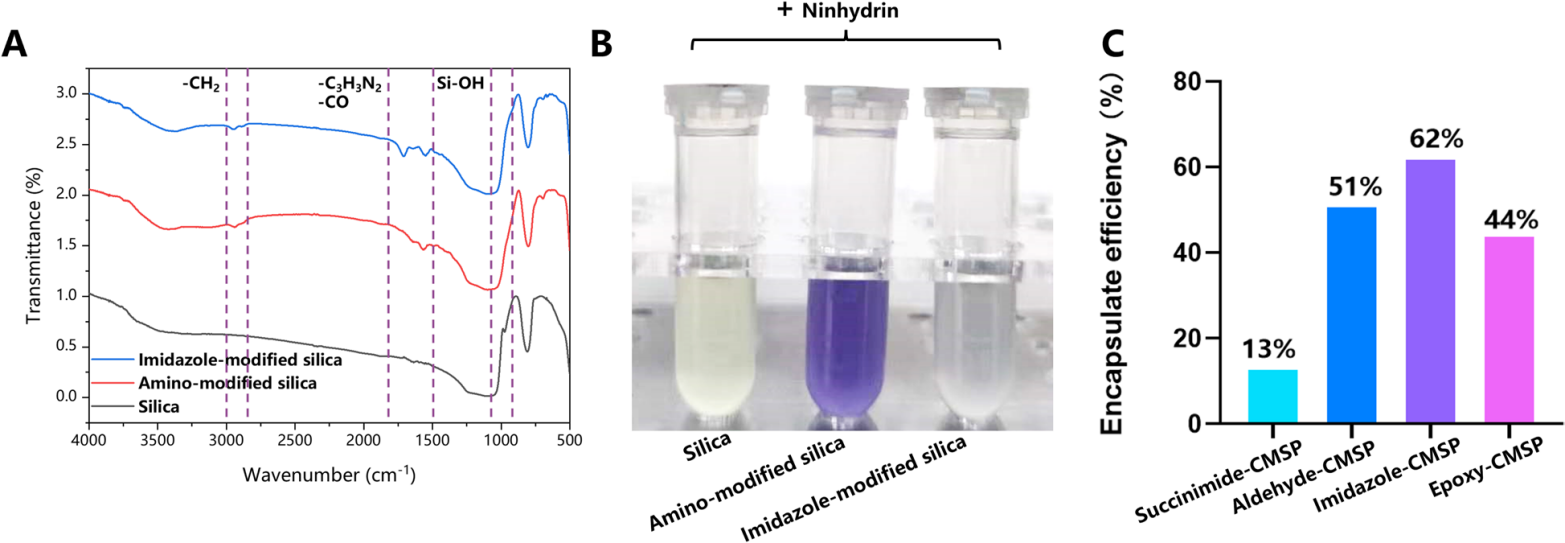
Infrared analysis (**A**) and chemical coloration (**B**) of imidazole- and (**C**) BCA protein assays of four different covalently-modified silica gel based CMSP

First, we design and synthesize succinimide-, aldehyde-, epoxy- and imidazole-modified silica gels. The functional groups on the surface of covalently modified silica gels were confirmed by infrared analysis and chemical coloration (Fig. 2, Fig. S1). Infrared analysis shows the absence of a silicon hydroxyl peak at 974 cm^−1^ and the appearance of a carbonyl-imidazole peak at 1709 cm^−1^ and 1641 cm^−1^ providing strong evidence that the imidazole group had been successfully attached to the silica gel surface (Fig. 2A). When the synthetic intermediate amino-modified silica gel was mixed with ninhydrin, it turned blue-purple indicating the presence of the amino group. The blue-purple faded when the amino group was replaced with an imidazole group or an aldehyde group (Fig. 2B). However, the coloration changes observed during the synthesis of succinimide- and epoxy-modified silica gels were less pronounced (Fig. S1). This could be attributed to the lower synthesized efficiency. The element rate analysis showed that C%, H%, and N% content of the imidazole- and aldehyde-modified silica gels were significantly higher than those of the other covalently modified silica gels (Table 1). This elemental content provided additional support for the successful attachment of the functional groups and the synthesized efficiency. Ultimately, the amount of protein in the supernatant was used to assess the binding efficiency of the modified silica gels to the cell membrane. As Fig. 2C shows, the imidazole- and aldehyde-modified silica-based CMSP exhibited a relatively high protein binding rate (up to 50%), in which the imidazole-modified was the highest (62%). This difference in binding efficiency could be attributed to the self-polymerization of glutaraldehyde, a known issue that can affect its binding properties [17, 18]. Thus, the imidazole-modified silica gel was identified as the most effective in terms of protein binding, which is applied in constructing H9c2/CMC with an extended lifespan and improved efficiency.

**Table 1:** The elemental composition on modified silica gel

	Elemental analysis (%)		
	C	H	N
Silica	–	–	–
Epoxy-modified silica	4.47	0.90	0.00
Succinimide-modified silica	3.66	0.60	0.34
Aldehyde-modified silica	11.30	1.61	1.30
Imidazole-modified silica	7.96	1.15	2.68

### Establishment and characterization of H9c2/CMC

To screen the myocardial protective compounds in YD, a H9c2/CMC was developed in this research. Previous studies have shown the presence of various membrane receptors, such as ion channels, on the cell membrane of H9c2 cardiac cell lines [19, 20]. Therefore, PPH, a blocker targeting calcium and sodium channels, was chosen as the positive control for quality assurance of the column. DXM, a hormone that specifically interacts with intracellular glucocorticoid receptors, served as the negative control. The selectivity, specificity, reproducibility, and stability of this H9c2/CMC were assessed according to previous studies [14].

#### Selectivity evaluation

**Fig. 3:**
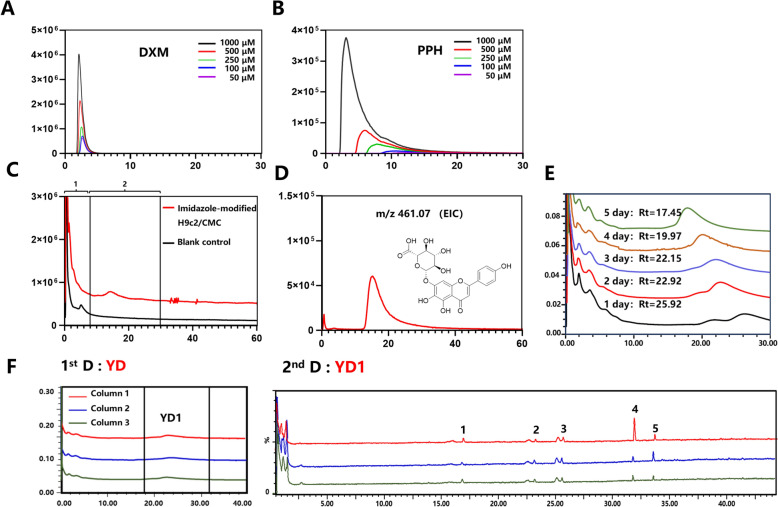
Method validation of H9c2/CMC, including selectivity (**A** and **B**), specificity (**C** and **D**), stability (**E**), and reproducibility (**F**)

As shown in Fig. 3A and B, the negative control DXM (1000 μM, 500 μM, 250 μM, 100 μM, 50 μM) exhibited minimal retention on the imidazole-modified H9c2/CMC column. In contrast, the retention time of PPH increased with decreasing concentration, indicating a marked concentration-dependent trend. These results indicated that the cell membrane in the column effectively retained active compounds.

#### Specificity evaluation

The specificity assessment of CMC was carried out by comparing the chromatographic peak and retention time of the imidazole-modified H9c2/CMC column to the blank control (imidazole-modified silica column). As shown in Fig. 3C, the first portion was a false positive component (0–8 min), which was assumed to be a portion of the component residing in the silica gel pore and flushed by the mobile phase, and the second portion was the possible active component (8 min later). The extract ion chromatogram of *m/z* 461.07 ([M−H]^−^ of scutellarin) was shown as a "trailing peak" (Fig. 3D), indicating the reliability of screening.

#### Stability evaluation

The activity of CMC columns decreases gradually during analysis because of the cell membrane exfoliation, resulting in a limited life span [16]. Thus, the stability of H9c2/CMC was assessed in regular and continuous use. The column’s life span was shown to be stable up to the fifth day, with the retention time decreasing to 17.45 min (from 25.92 min), which is approximately twice as long as the non-covalent CMC (about 60 h, as reported by 14). Therefore, further screening experiments could be facilitated within a 5-day period (Fig. 3E).

#### Reproducibility evaluation

The reproducibility of CMC was assessed by examining the chromatographic peak and retention time in the 1^st^ D and 2^nd^ D spectra acquired from three parallel CMC columns. As shown in Fig. 3F, the spectra of 1^st^ D and 2^nd^ D are almost identical, with the relative standard deviations (RSDs) of retention time and peak area of YD being 2.23% and 8.40%, respectively (Table S1), indicating satisfactory reproducibility of the imidazole-modified H9c2/CMC.

### High-content components knockout strategy for screening active compounds in YD

According to the prior quantitative analysis [15], the contents of various compounds in YD varied significantly, with certain compounds present at concentrations exceeding 0.8 mg·g^−1^. These high-content compounds often cause column overload and potentially overshadow the screen of other activity compounds.

To address this issue, we implemented a slightly modified approach of the high-content components knockout strategy. In the first round, the original YD solution was directly prepared and applied for screening, enabling the identification of potential bioactive compounds with high contents in YD. Subsequently, the K_*(x)*_YD was employed for the second-round screening. Here, *x* represents one of the intermediates, and K_*(x)*_ indicates the removal of *x* from YD. Finally, each individual intermediate was examined in the third-round screening, allowing us to eliminate the high-content components and specifically target activity compounds. This approach allowed us to gain a more comprehensive understanding of the activity compounds in YD.

#### The first round of screening

**Fig. 4:**
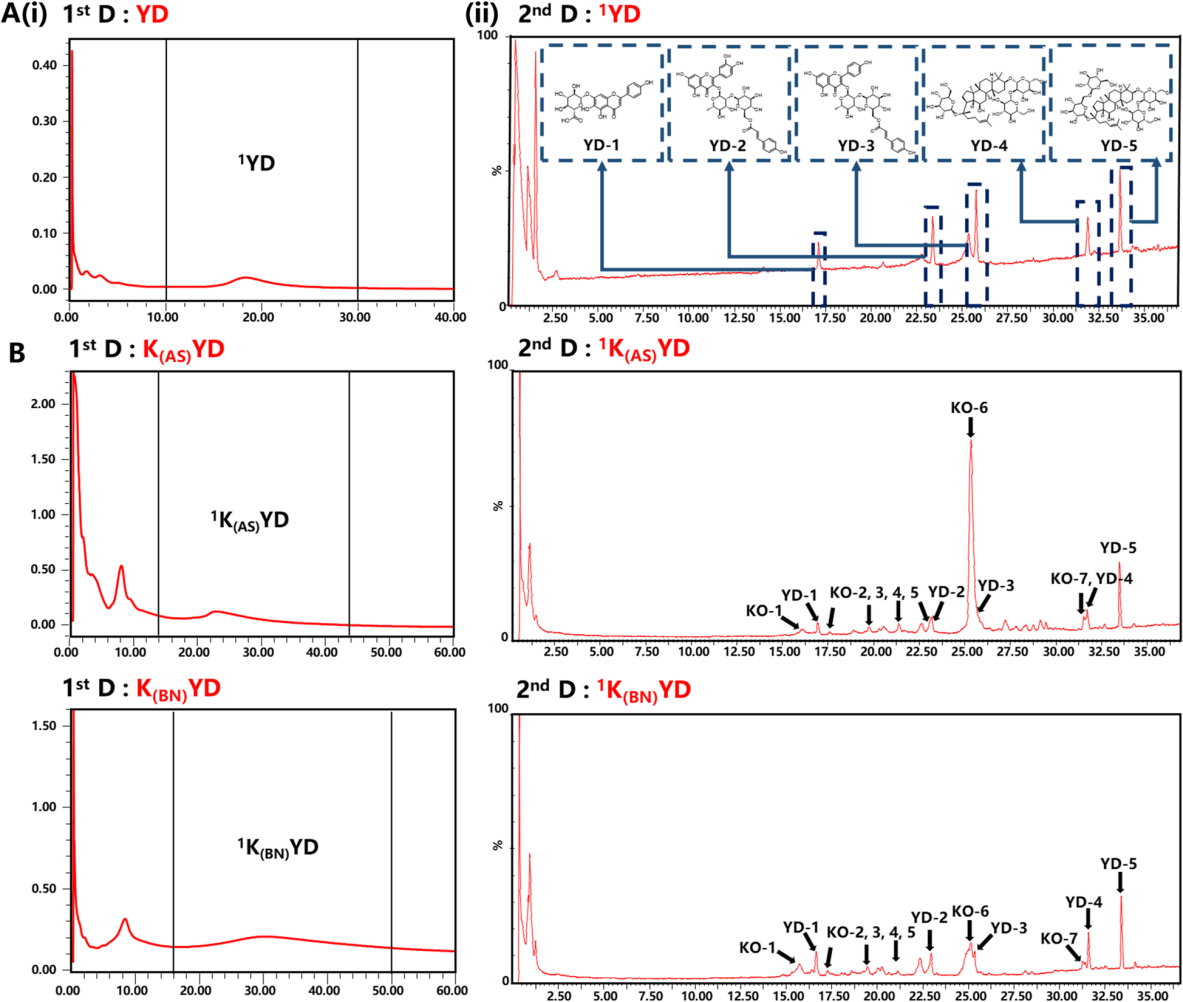
2D chromatography of YD original solutions (**A**), K_*(AS)*_YD and K_*(BN)*_YD (**B**) by the 2D H9c2/CMC-RPLC/MS system

The chromatogram of YD original solutions on the CMC column is displayed in Fig. 4A(i). Subsequently, the potential active compounds, ^1^YD eluting from 10 to 30 min, were then introduced into the second dimension (2^nd^D). As shown in Fig. 4A(ii), five peaks were identified according to accurate mass data and fragment ion information from QTOF MS analysis. The identified peaks included scutellarin (YD-1), quercetin 3-*O-*2ʺ-(6ʺ-p-coumaroyl) glucosyl rhamnoside (YD-2), kaempferol 3-*O-*2ʺ-(6ʺ-p-coumaroyl) glucosyl rhamnoside (YD-3), ginsenoside Rb1 (YD-4), and ginsenoside Rd (YD-5). YD-1 prevented myocardial ischemia-reperfusion injury by inhibiting NLRP3 inflammasome activation [21]. YD-4 reduced ischemia-reperfusion injury by inhibiting cardiomyocyte autophagy [22]. YD-5 promoted cardiac repair after a myocardial infarction by modulating monocytes/macrophages subsets conversion [23]. YD-2 and YD-3 exhibited antioxidant properties, yet reports of myocardial protection remain absent. As anticipated, the screened compounds showed relatively high content in YD (≥ 0.840 mg·g^–1^) except ginsenoside Rd (0.017 mg·g^–1^).

#### The second round of screening

Volatile compounds from AS and BN are commonly detected using GC-MS but challenged by using LC-MS. Directly profiling the bioactivity of AS and BN using the established 2D H9c2/CMC-RPLC-MS system is not feasible. However, through the analysis of AS and BN-knockout samples, their contributions to myocardial effect in YD can be revealed by comparing them with the original YD. The screening results of K_*(AS)*_YD and K_*(BN)*_YD were shown in Fig. 4B. After knocking out AS and BN, respectively, seven potential active compounds were identified in both cases, including rutin (KO-1), salvianolic acid H/J/I (KO-2), salvianolic acid G (KO-3), isochlorogenic acid B (KO-4), isosalvianolic acid A (KO-5), salvianolic acid B (KO-6), and apigenin (KO-7). Notably, salvianolic acid B (KO-6) was more readily identified after AS-knockout, while rutin (KO-1) was notably present after BN-knockout. These findings indicated that AS and BN play a crucial role in the myocardial protective effects of YD, with distinct interactions.

Moreover, we sequentially knocked out other intermediates and introduced the recombinant YD samples for further screening. A total of seven potential active compounds were screened out (Fig. S2A). Among these, salvianolic acid B (KO-6) was reported to regulate autophagy in myocardial ischemia, inhibiting cardiomyocyte apoptosis [24]. Apigenin (KO-7) was observed to mediate pyroptosis and apoptosis, offering protection against myocardial injury induced by ischemia or hypoxia [25].

#### The third round of screening

Furthermore, to supplement our understanding of active compounds, the individual intermediates were screened using the 2D H9c2/CMC-RPLC-MS system (Fig. S2B). This supplementary evaluation unveiled an additional 12 potential active compounds. Specifically, C-3 mitigated cardiac fibrosis after long-term pressure overload [26], whereas C-7 protected H9c2 cardiomyocytes against apoptosis induced by hypoxia and reoxygenation [27]. Their concentrations in YD capsule ranged from 0.073 to 0.323 mg/g. In conclusion, after three rounds screening, 24 potential myocardial protective active compounds were identified, including 8 flavonoids, 8 phenolic acids, 4 saponins, and 4 tanshinones (Table 2, Fig. S3).

**Table 2:** Potential active compounds characterized by H9c2/CMC-RPLC/MS system

NO.	2^nd^ RT (min)	Formula	Precursor ions (*m/z*)	Diff.	Main fragment ions (*m/z*)	Identification
KO-1	16.05	C_27_H_30_O_16_	609.1484 [M−H]^−^	− 3.76	301.03, 283.03	Rutin
YD-1	16.70	C_21_H_18_O_12_	461.0707 [M−H]^−^	4.00	285.04, 179.03	Scutellarin*
KO-2	17.65	C_27_H_22_O_12_	537.1021 [M−H]^−^	3.25	295.06, 185.02	Salvianolic acid H/J/I
KO-3	19.75	C_18_H_12_O_7_	339.0491 [M−H]^−^	5.66	280.04, 252.04	Salvianolic acid G
C-1	19.81	C_20_H_18_O_10_	417.0831 [M−H]^−^	− 0.91	197.04, 175.04	Isosalvianolic acid D
C-2	20.49	C_21_H_18_O_11_	445.0776 [M−H]^−^	0.08	269.05, 175.03	Apigenin-7-*O*-glucuronide
KO-4	21.45	C_25_H_24_O_12_	515.1177 [M−H]^−^	3.49	353.09, 179.03	Isochlorogenic acid B
C-3	21.89	C_18_H_16_O_8_	359.0771 [M−H]^−^	0.39	179.03, 161.02	Rosmarinic acid
C-4	22.08	C_21_H_20_O_11_	447.0903 [M−H]^−^	6.65	285.04, 255.03	Kaempferol 3-*O*-glucoside
C-5	22.76	C_37_H_38_O_19_	785.1915 [M−H]^−^	2.48	609.15, 151.03	Kaempferol 3-*O*-acyldiglycoside
KO-5	23.01	C_26_H_22_O_10_	493.1150 [M−H]^−^	− 1.98	295.06, 185.02	Isosalvianolic acid A
YD-2	23.09	C_36_H_36_O_18_	755.1836 [M−H]^−^	− 0.94	609.14, 300.03	Quercetin 3-*O-*2ʺ-(6ʺ-p-coumaroyl) glucosyl rhamnoside
KO-6	25.35	C_36_H_30_O_16_	717.1434 [M−H]^−^	3.77	519.09, 321.04	Salvianolic acid B*
YD-3	25.45	C_36_H_36_O_17_	739.1859 [M−H]^−^	2.80	593.15, 284.03	Kaempferol 3-*O-*2ʺ-(6ʺ-p-coumaroyl) glucosyl rhamnoside*
C-6	30.11	C_26_H_20_O_10_	491.0964 [M−H]^−^	4.00	196.91, 174.95	Isosalvianolic acid C
KO-7	31.54	C_15_H_10_O_5_	269.0435 [M−H]^−^	7.58	211.04, 181.06	Apigenin
YD-4	31.80	C_54_H_92_O_23_	1107.6006 [M−H]^−^	− 4.45	945.56, 783.48	Ginsenoside Rb1*
YD-5	33.67	C_48_H_82_O_18_	991.5554 [M + COOH]^+^	− 7.48	945.55, 621.43	Ginsenoside Rd*
C-7	37.30	C_36_H_60_O_8_	665.4247 [M + COOH]^+^	3.74	619.42, 161.04	Ginsenoside Rk3
C-8	37.40	C_36_H_60_O_8_	665.4303 [M + COOH]^+^	− 4.96	619.42, 161.04	Ginsenoside Rh4
C-9	39.31	C_19_H_16_O_4_	309.1121 [M + H]^+^	0.12	263.10, 235.07	Tanshinaldehyde
C-10	41.40	C_18_H_14_O_3_	279.0993 [M + H]^+^	8.17	261.09, 205.10	Dihydrotanshinone I
C-11	43.10	C_20_H_18_O_5_	339.1220 [M + H]^+^	2.07	233.09, 205.10	Methyl tanshinone
C-12	44.84	C_19_H_20_O_3_	297.1467 [M + H]^+^	6.15	268.11, 251.14	Cryptotanshinone

^*^Identification was confirmed with reference standards

### Investigating the mechanisms of drug action of YD in myocardial protection

#### Bioinformatics analysis for identification of the core target

**Fig. 5:**
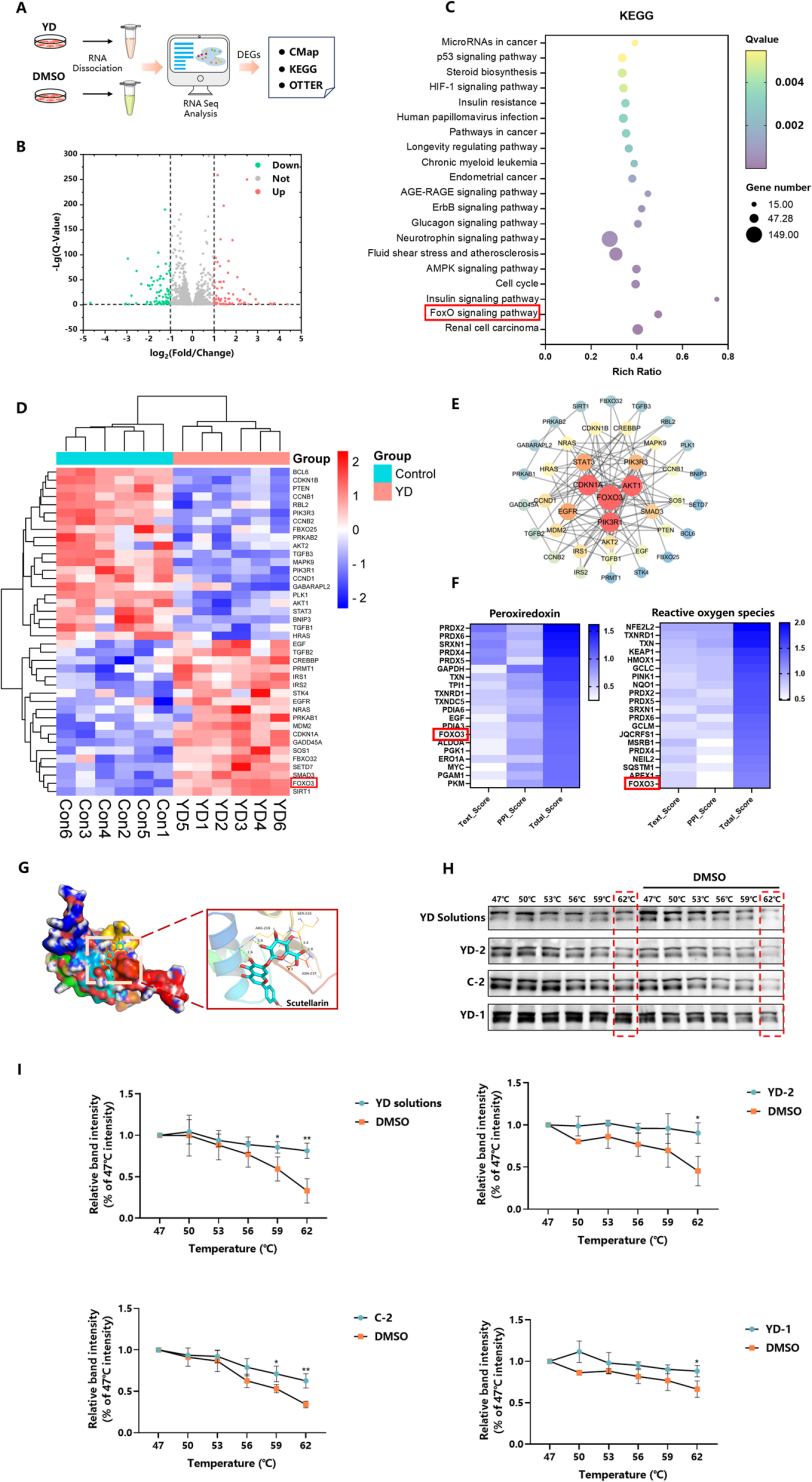
Overview of the strategy used in MCF-7 cells (**A**), the differential gene expression volcano map (**B**), KEGG enrichment analysis (**C**), the differential gene expression heatmap of FOXO signaling pathway (**D**), and the visualization of FOXO signaling pathway through PPI network (**E**). The target enrichment of OTTER (**F**). Molecular docking of scutellarin and FOXO3 (**G**). The results of cellular thermal shift assay (**H** and **I**), * P < 0.05, ** P < 0.01

To investigate the potential pharmacological mechanism of YD (Fig. 5A), we utilized the online Connectivity Map database (CMap) (https://www.broadinstitute.org/connectivity-map-cmap) [28]. Since the gene expression data recorded in CMap was sourced from the MCF-7 or Hela cell line, we gathered the up-regulated and down-regulated gene information from YD-tread MCF-7 cells. As a result, 1722 genes were up-regulated while 1766 genes were down-regulated following YD treatment in comparison to the control group (Fig. 5B, Table S3). These differentially expressed genes (DEGs) were then compared with those in CMap. The analysis indicted that the gene expression regulated by YD is similar to that of EGFR inhibitor, sodium channel blocker, and MAP kinase inhibitor (Table S2). Among them, parthenolide has been shown to ameliorate myocardial injury by downregulating the expression of antioxidant enzymes [29]; isoliquiritigenin exhibits myocardial protective effects by reducing reactive oxygen species (ROS) during hypoxia/reoxygenation processes [30]; quinidine exerts its antiarrhythmic effects by inhibiting the transmembrane movement of sodium ions [31], while piperlongumine exhibits an anti-myocardial fibrosis effect and induce ROS level [32]. According to the CMap analysis results, it appears that the regulation of oxidative stress constitutes one of the mechanisms through which YD provides myocardial protection.

Subsequently, KEGG pathway analysis was performed using the DEGs form YD treatment. The KEGG enrichment analysis revealed that YD regulates p53, HIF-1, and FOXO signaling pathways (Fig. 5C). Among them, the FOXO pathway plays a role in regulating oxidative stress and impacting myocardial function [33]. We identified 40 DGEs involved in the FOXO pathway, among which FOXO3 is notably up-regulated following YD treatment (Fig. 5D). Consequently, a protein–protein interaction (PPI) network was constructed based on the DEGs within the FOXO signaling pathway. This PPI network highlighted FOXO3, a primary subtype of FOXO in the cardiac tissue and a crucial therapeutic target for heart disease [34], as a significant target protein regulated by YD (Fig. 5E). Additionally, the novel computational tool, OTTER (http://otter-simm.com/otter.html) [35], was performed for discovery the related proteins associated with oxidative stress regulating by YD. We highlighted peroxiredoxin and reactive oxygen species (ROS) activity as critical factors in oxidative stress of myocardial protection [36, 37]. As shown in Fig. 5F, FOXO3 was also recognized as the principal target both correlating with peroxiredoxin and ROS activity based on the DEGs from YD. FOXO3, a transcription factor that belongs to the forkhead family, serves as a key regulator in the heart, which can relieve heart inflammation and promote heart repair [38, 39]. Therefore, we proposed FOXO3 as a potential key target for the myocardial protective effects of YD.

#### Molecular docking and experimental assessment of the binding affinity

Subsequently, the 24 potentially active compounds screened using H9c2/CMC-RPLC-MS method were followed by molecular docking analysis with the FOXO3 protein. Generally, a binding affinity of less than − 5.0 kcal/mol suggests that these compounds interact with the target protein [40–42]. The docking results revealed that all 24 compounds exhibit binding affinities with FOXO3 below − 5.0 kcal/mol, specifically ranging from − 7.5 to − 5.6 kcal/mol (Table 3, Fig. 5G). In addition, a lower binding energy indicated a stronger interaction between the ligand and the target protein. Notably, among the 24 potentially active compounds, 9 exhibited lower binding energies than quinidine, an antiarrhythmic agent, which has a binding energy of − 6.7 kcal/mol.

**Table 3:** Molecular docking results of 24 compounds and quinidine

Name	Hydrogen bond	Binding energy (Kcal/mol)	Source
Rutin	6	− 6.7	GF
Scutellarin	5	− 7.3	EH
Salvianolic acid H/J/I	9	− 6.3	SM
Salvianolic acid G	5	− 6.8	SM
Isochlorogenic acid B	10	− 5.6	EH
Isosalvianolic acid D	5	− 6.6	SM
Apigenin-7-*O*-glucuronide	3	− 7.4	EH
Rosmarinic acid	5	− 5.8	SM
Kaempferol 3-*O*-glucoside	4	− 5.9	GF
Kaempferol 3-*O*-acyldiglycoside	4	− 5.6	GF
Isosalvianolic acid A	8	− 6.3	SM
Quercetin 3-*O-*2ʺ-(6ʺ-p-coumaroyl) glucosyl rhamnoside	5	− 7.5	GF
Salvianolic acid B	6	− 5.7	SM
Kaempferol 3-*O-*2ʺ-(6ʺ-p-coumaroyl) glucosyl rhamnoside	2	− 6.6	GF
Isosalvianolic acid C	3	− 6.6	SM
Apigenin	0	− 6.2	EH
Ginsenoside Rb1	10	− 6.3	Mix
Ginsenoside Rd	2	− 5.9	Mix
Ginsenoside Rk3	6	− 7.0	Mix
Ginsenoside Rh4	6	− 6.9	Mix
Tanshinaldehyde	1	− 6.3	SM
Dihydrotanshinone I	0	− 6.8	SM
Methyl tanshinonate	1	− 6.5	SM
Cryptotanshinone	1	− 6.8	SM
Quinidine	1	− 6.7	

To further validate the relationship between FOXO3 and YD, we selected the YD solutions and the top three compounds ranked by molecular docking with the H9c2 protein solutions to perform the Cellular Thermal Shift Assay (CETSA). The results demonstrated that YD solutions, YD-1, YD-2, and C-2, could enhanced the thermal stability of FOXO3 compared to the DMSO (Fig. 5H, I), respectively. It suggested these compounds interacted with FOXO3 and strongly indicated that FOXO3 may represent a key target for YD myocardial protection.

## Conclusion

In this work, a highly efficient imidazole-modified silica gel was synthesized, exhibiting a high protein binding capacity. Subsequently, the imidazole-modified silica gel H9c2/CMC was constructed, demonstrating excellent selectivity, specificity, stability, and reproducibility. Then, the 2D H9c2/CMC-RPLC-MS system was established and applied for screening of myocardial protective active compounds from YD. Through integration with a high-content components knockout strategy, 24 potential myocardial protective compounds were identified, comprising 8 flavonoids, 8 phenolic acids, 4 saponins, and 4 tanshinones. Bioinformatics analysis, utilizing RNA-seq, indicated that the FOXO signaling pathway plays a significant role in the cardioprotective effects of YD. All screened potential active compounds exhibit strong binding affinities with FOXO3, as evaluated by molecular docking. The present work significantly enhances the application of CMC and provides a comprehensive pharmacodynamic foundation for screening activity compounds from CMs. Furthermore, the 2D imidazole-modified CMC-RPLC-MS system can be used with other cell lines to effectively separate pharmacodynamic effects in complex mixtures.

## Supplementary Information

Supplementary Material 1.

Supplementary Material 2.

## Data Availability

The datasets used and/or analyzed during the current study are available from the corresponding author on reasonable request.

## Abbreviations

CMC: Cell membrane chromatography
CMs: Chinese medicines
YD: Yindan Xinnaotong soft capsule
2D-LC: Two-dimensional liquid chromatography
RPLC: Reverse phase liquid chromatography
CMSP: Cell membrane stationary phase
CDI: 1,1-Carbonyldiimidazole
GA: Glutaraldehyde
GLYMO: (3-Glycidyloxypropyl) trimethoxysilane
GF: Ginkgo Folium
ESM: Ethanol extract of Salviae Miltiorrhizae Radix et Rhizoma
WSM: Water extract of Salviae Miltiorrhizae Radix et Rhizoma
Mix: Mixed extract of Notoginseng Radix et Rhizoma, Crateagi Fructus, and Gynostemma pentaphyllum
EH: Water extract of Erigerontis Herba
BN: Borneolum
AS: Oil of Allii Sativi Bulbus
DXM: Dexamethasone acetate
PPH: Propafenone hydrochloride
