# Supplementary material for: Two-dimensional cell membrane chromatography guided screening of myocardial protective compounds from Yindan Xinnaotong soft capsule
Source: Chin Med. 2025 Jan 4;20:5. doi: 10.1186/s13020-024-01046-1 (PMC11700464; doi:10.1186/s13020-024-01046-1)
Supplement: Supplementary file 1 — Supplementary Material 1. [file 13020_2024_1046_MOESM1_ESM.docx]

**Supplementary information**

**Two-dimensional cell membrane chromatography guided screening of myocardial protective compounds from Yindan Xinnaotong soft capsule**

Si-Min Shao, Xuan Ji, Xing Wang, Run-Zhou Liu, Yu-Ru Cai, Xiaobing Lin, Ze-Jie Zeng, Ling Chen, Liu Yang, Hua Yang*, Wen Gao*

*State Key Laboratory of Natural Medicines, School of Traditional Chinese Pharmacy, China Pharmaceutical University, No. 639 Longmian Road, Nanjing 211198, China*

*Corresponding author. Tel.: +86-25-8618-5219.

E-mail address: [gw_cpu@126.com](mailto:gw_cpu@126.com) (W. Gao); [yanghuacpu@126.com](mailto:yanghuacpu@126.com) (H. Yang)**Figure**

**Fig. S1.** Result of infrared analysis of succinimide-modified silica gel (A), aldehyde-modified silica gel (B), epoxy-modified silica gel (C), and their chemical coloration (D).

**Fig. S2.** 2D chromatography of K*_(x)_*YD samples (A) and the individual intermediates (B) by the 2D H9c2/CMC-RPLC/MS system.

**Fig. S3.** Chemical structures of 24 potential myocardial protective components.

**Table**

**Table S1.** Reproducibility of H9c2/CMC columns.

**Table S2.** CMap analysis results.

**Table S3.** The RNA-seq results of FPKM and differentially expressed genes.**Figure**


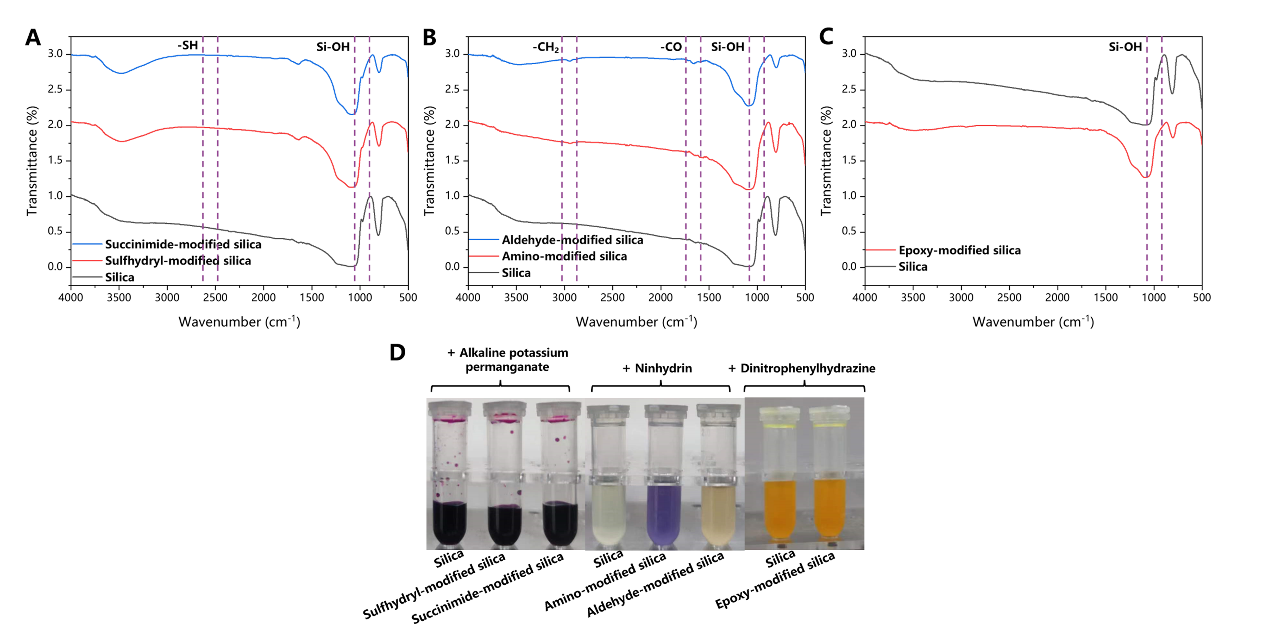


**Fig. S1.** Result of infrared analysis of succinimide-modified silica gel (A), aldehyde-modified silica gel (B), epoxy-modified silica gel (C), and their chemical coloration (D).


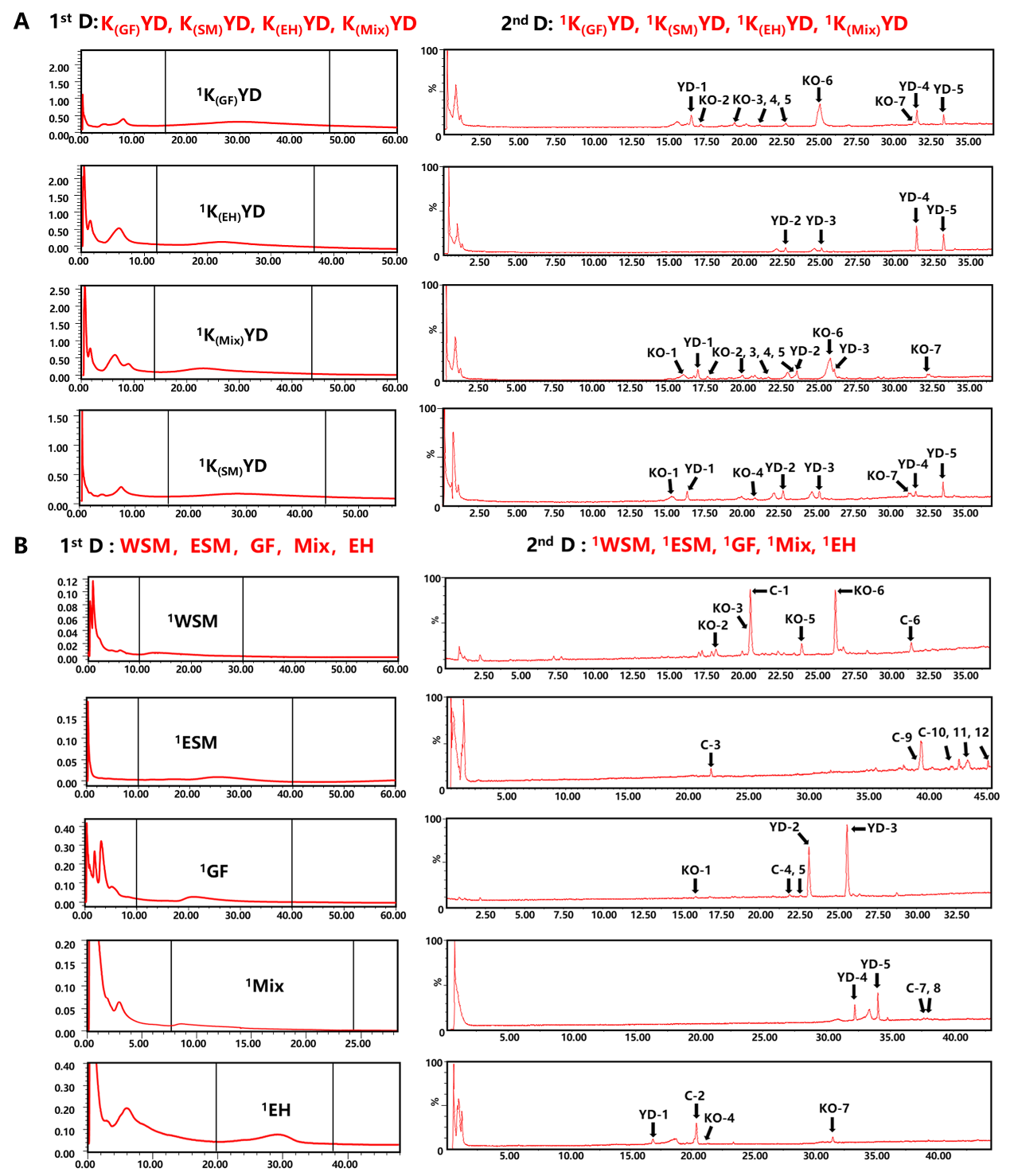


**Fig. S2.** 2D chromatography of K*_(x)_*YD samples (A) and the individual intermediates (B) by the 2D H9c2/CMC-RPLC/MS system.


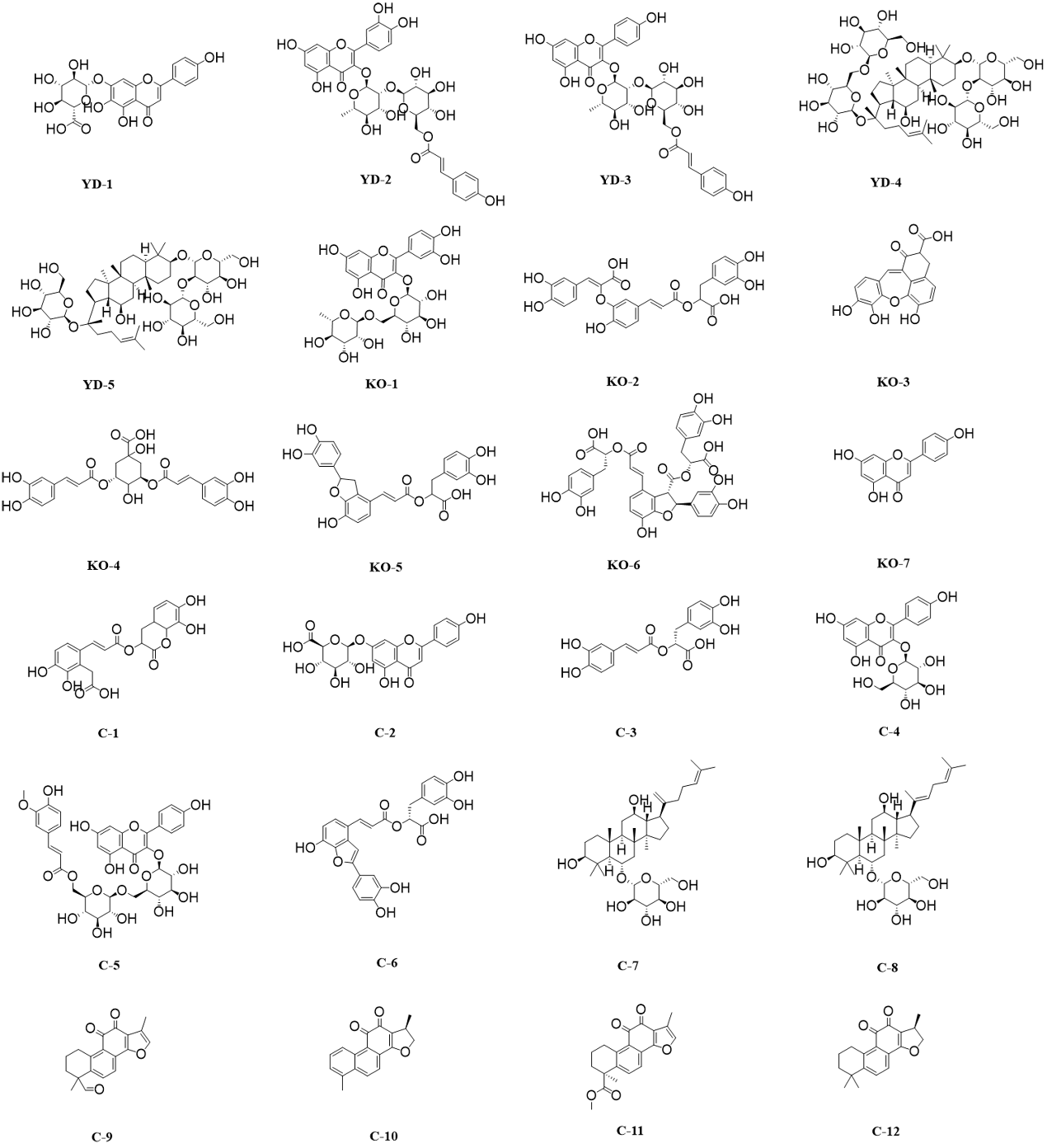


**Fig. S3.** Chemical structures of 24 potential myocardial protective components.

**Table**

**Table S1.** Reproducibility of H9c2/CMC columns

| YD | CMC columns | | | RSD (%) |
| --- | --- | --- | --- | --- |
|  | Column I | Column II | Column III |  |
| Retention time (min) | 22.922 | 23.492 | 23.965 | 2.23 |
| Peak area (mAU·s) | 3753050 | 3915240 | 4402683 | 8.40 |

**Table S2.** CMap analysis results

| No. | Score | Component | MOA |
| --- | --- | --- | --- |
| 1 | 99.14 | Arvanil | TRPV agonist |
| 2 | 99.08 | Securinine | GABA receptor antagonist, TP53 activator |
| 3 | 98.12 | Parthenolide | NFkB pathway inhibitor, Adiponectin receptor agonist |
| 4 | 97.69 | methyl-2,5-dihydroxycinnamate | EGFR inhibitor, Tyrosine kinase inhibitor |
| 5 | 97.14 | Prostaglandin | Prostanoid receptor antagonist |
| 6 | 96.69 | Isoliquiritigenin | Guanylate cyclase activator |
| 7 | 94.67 | Quinidine | Sodium channel blocker |
| 8 | 92.29 | Rottlerin | MAP kinase inhibitor, Protein kinase inhibitor |
| 9 | 91.99 | Piperlongumine | Glutathione transferase inhibitor |
| 10 | 91.2 | Withaferin A | IKK inhibitor |

**Preparation of aldehyde-modified silica gel**

Make appropriate adjustments according to laboratory conditions [1]. First, the silica gel was decorated with APTES to obtain an amino group on the surface. In brief, 0.2 g of degassed silica gel was mixed with 0.1 mL of APTES in 10 mL of toluene under an inert argon atmosphere at 110 °C for 12 h. Then the sample was dried and suspended in 100 mL of glutaraldehyde (5 wt %) diluted in methanol and shaken for 2 h under room temperature for the binding of glutaraldehyde onto APTES. At last, the other end of glutaraldehyde would be able to link to cell membranes by reacting with the abundant amino groups on the membranes after 5 min of vortex in vacuum and 24 h of incubation at 4 °C.

**Preparation of succinimide-modified silica gel**

Make appropriate adjustments according to laboratory conditions [2]. First, the silica gel was decorated with MPTS to obtain a sulfhydryl group on the surface. In brief, 1 g of degassed silica gel was mixed with 1 mL of MPTS in 100 mL of DMF under an inert argon atmosphere at 60°C for 5 h. Then the sample was dried and suspended in 500 mL DMSO of 5% GMBS and shaken for 2 h under room temperature for the binding of GMBS onto MPTS. At last, the other end of GMBS would be able to link to cell membranes by reacting with the abundant amino groups on the membranes after 5 min of vortex in vacuum and 24 h of incubation at 4 °C.

**Preparation of epoxy-modified silica gel**

Make appropriate adjustments according to laboratory conditions [3]. First, the silica gel was decorated with GLYMO to obtain an epoxy group on the surface. In brief,1 g of degassed silica gel was mixed with 10 mL of GLYMO in 90 mL of toluene under an inert argon atmosphere at 110 °C for 8 h. At last, the end of GLYMO would be able to link to cell membranes by reacting with the abundant amino groups on the membranes after 5 min of vortex in vacuum and 24 h of incubation at 4 °C.

**References**

[1] Ding X., Cao Y., Yuan Y., et al, 2016. Development of APTES-Decorated HepG2 Cancer Stem Cell Membrane Chromatography for Screening Active Components from Salvia miltiorrhiza. Anal Chem. 88 (24), 12081-12089. <https://doi.org/10.1021/acs.analchem.6b02709>.

[2] Gu Y., Chen X., Wang Y., et al, 2020. Development of 3-mercaptopropyltrimethox-ysilane (MPTS)-modified bone marrow mononuclear cell membrane chromatography for screening anti-osteoporosis components from Scutellariae Radix. Acta Pharm Sin B. 10 (10), 1856-1865. <https://doi.org/10.1016/j.apsb.2020.01.019>.

[3] Sun J., Wei L., Wang Y., et al, 2018. Immobilization of carbonic anhydrase on polyvinylidene fluoride membranes. Biotechnol Appl Biochem. 65 (3), 362-371. <https://doi.org/10.1002/bab.1629>.
